# An existential model of addiction

**DOI:** 10.1192/j.eurpsy.2021.1323

**Published:** 2021-08-13

**Authors:** G. Grech

**Affiliations:** Psychiatry, Mount Carmel Hospital, Attard, Malta

**Keywords:** authenticity, being-with-drug, Addiction, existentialism

## Abstract

**Introduction:**

Despite existentialism positing that existential concerns are universal, research into the existential issues related to addiction remains scarce. An existential model of addiction is lacking.

**Objectives:**

This research aims to develop an existential model of addiction, conceptualising the development of addiction through to authenticity.

**Methods:**

A scoping literature review was carried out using PUBMED, reference lists and internet websites.

**Results:**

Psychopathology, from an existential point of view, occurs as a result of the avoidance of the existential givens which are death, freedom, existential isolation and meaninglessness. In this model, addiction is positioned as a coping mechanism to deal with the existential or neurotic anxiety which arises from facing or avoiding the existential givens. Addiction is defined as being-with-drug; a state in which our inherent relation to others is replaced by a relation with a substance. This state is understood from the ontological, axiological, ethical and praxeological levels, shedding light on the phenomenological experience of addiction. The existential dilemmas around meaning, loneliness, death, freedom, guilt and control while living with addiction are discussed. Finally, existential crises, boundary situations and secondary suffering are seen as the main motivators to overcome addiction.

**Conclusions:**

Phenomenological and existential research support the fact that existential issues are relevant to addiction. This model explains the relationships between existential concepts and addiction, while providing a framework for clinicians to explore and address these issues with patients.

